# Prediction of PTSD related to COVID-19 in emergency staff based on the components of self-compassion and perceived social support

**DOI:** 10.1186/s12888-022-04017-8

**Published:** 2022-05-31

**Authors:** Kavous Shahsavarinia, Parastoo Amiri, Zahra Mousavi, Neda Gilani, Mohammad Saadati, Hassan Soleimanpour

**Affiliations:** 1 Road Traffic Injury Research Center, Tabriz University of Medical Sciences, Tabriz, Iran; 2 Emergency and Trauma Care Research Center, Tabriz University of Medical Sciences, Tabriz, Iran; 3 Research Center of Psychiatry and Behavioral Science, Tabriz University of Medical Sciences, Tabriz, Iran; 4 Department of Statistics and Epidemiology, Faculty of Health, Tabriz University of Medical Sciences, Tabriz, Iran; 5 Khoy University of Medical Sciences, Khoy, Iran

**Keywords:** COVID-19, Self-compassion, Perceived social support, PTSD, Emergency staff

## Abstract

**Background:**

On March 11^th^, 2020, the World Health Organization (WHO) proclaimed Coronavirus Disease of 2019 **(**COVID-19) a pandemic. In addition to severe health problems, the disease has had a major psychological impact on the public. The aim of this research was to examine the association between Post-Traumatic Stress Disorder (PTSD) related to COVID**-**19 in emergency staff and self-compassion and perceived social support.

**Methods:**

Data were collected from 222 emergency staff working in two referral educational and health centers for COVID-19 affiliated to Tabriz University of Medical Sciences. The participants were recruited six months following the first case of hospitalization for COVID-19 in these two hospitals in Tabriz, Iran. Four questionnaires were used to measure the variables, including a researcher-made demographic checklist, PTSD Checklist for DSM-5 (PCL-5), the Multidimensional Perceived Social Support Scale (MSPSS) and the Self-Compassion Scale.

**Results:**

The findings showed that age (*r* = 0.17, *P* = 0.034), self-judgment (*r* = 0. 36, *P* < 0.001), isolation (*r* = 0.44, *P* < 0.001) and over-identification (*r* = 0.15, *P* = 0.031) were associated with PTSD score, and there was also a statistically significant inverse relationship between the score of the self-kindness (*r* = - 0.19, *P* = 0.006) subscale and the overall score of PTSD in the emergency staff.

**Conclusion:**

During the COVID-19 pandemic, emergency staff have persistently faced potentially traumatic situations as first-line healthcare workers, suggesting the direness of this group’s mental health. By identifying the predisposing factors of the psychological pathology under study, this research can be applied in clinical practice and provide useful information for designing special interventions and protocols for emergency staff.

**Supplementary Information:**

The online version contains supplementary material available at 10.1186/s12888-022-04017-8.

## Background

Lower respiratory infections remain the deadliest infectious disease worldwide [1]. In December 2019, a severe acute infectious respiratory syndrome caused by a new coronavirus (SARS-CoV-2) was identified in Wuhan, China. On March 11^th^, 2020, the World Health Organization (WHO) proclaimed coronavirus disease 2019 (COVID-19) a pandemic [2]. In addition to severe medical problems, this disease has also had severe psychological effects on people and healthcare workers (HCW_s_).[3].According to previous investigations from severe acute respiratory syndrome (SARS) or Ebola epidemics, the onset of a sudden and immediately life-threatening illness could lead to terrific amounts of pressure on HCWs [4]. The level and type of psychological effect on individuals during an epidemic can vary extensively depending on the degree of exposure experienced [3]. HCWs are more at risk for psychological problems due to direct exposure to the disease. As in the SARS epidemic, patients with SARS and HCWs who cared for them reported the greatest emotional distress [5, 6]. One of the psychological disorders that treatment staff may experience is post-traumatic stress disorder (PTSD). The COVID-19 pandemic highlights the vital need to focus on the mental health of HCWs involved in responding to this emergency. It has consistently been shown that a high proportion of HCWs are at a higher risk for developing PTSD [7, 8].

In a meta-analysis and systematic review, the frequency of PTSD in HCWs exposed to SARS/ Middle East Respiratory Syndrome (MERS) /COVID-19 was 20.7% [9]. In this meta-analysis and systematic review, which examined 115 individual studies covering 60,458 individuals, SARS/MERS/COVID-19 had a substantial impact on HCWs’ physical and mental health [9]. Meanwhile, HCWs are even more exposed to psychiatric symptoms than patients. The frequency of other psychiatric disorders such as anxiety and depression were 29% and 26.3%, respectively, in HCWs compared to the general population exposed to SARS and MERS, for whom the figures were 14.8% and 15% [9, 10].

As of August 9^th^, 2021, there were around 203 million confirmed cases of COVID-19, including 4.3 million deaths. Harmful psychological effects are expected to be long-lasting in vulnerable groups, especially among frontline HCWs, given the extent of the crisis they experience. While experience hard quarantine and social distancing measures is a necessary strategy to curb the spread of the virus, it has also had a major negative impact on mental health indicators; the long-term consequences of these measures are yet to be assessed on a global scale [11]. Since the onset of the COVID-19 pandemic, research on psychological traumas related to COVID-19 has shown HCWs to be one of the most vulnerable groups during this time. Various studies have shown that HCWs experience a variety of stress-related disorders, including PTSD, during the COVID-19 pandemic. Other studies have investigated the psychological and demographic factors affecting the incidence of PTSD in HCWs [7, 9, 12–14]. Due to the prolongation of the pandemic, these findings become even more important. HCWs around the world have been providing services to patients with COVID-19 for nearly two years, and psychological disorders that they develop do not only harm themselves, but also affect their job performance and the quality of healthcare services.

Perceived social support is one of the most significant protective factors in trauma-related disorders. A possible explanation may be offered by the ‘‘buffering hypothesis’’, according to which social support received during times of intense stress may reduce the psychological impact of traumatic events [15]. Low social support is a probable risk factor for PTSD in the COVID-19 pandemic [16]. This relationship becomes even more important when noting that the nature of COVID-19 is such that it leads to social isolation and deprivation of social support, especially in HCWs. This imposed social isolation and loneliness can take a toll in the long run [16]. An analytical cross-sectional study was conducted on 85 HCW’s at PAF hospital to assess the frequency of depression, anxiety, stress, and their association with perceived social support among doctors. The results showed that stress was the most common mental health issue among HCWs (physicians in Pakistan) and high social support positively affected this group’s mental health during the pandemic. Social support can prevent long-term issues such as PTSD and preserve psychological and mental health in HCWs [17, 18].

Another concept that has been explored in emotional disorders such as PTSD is self-compassion. According to Neff, self-compassion represents a balance between increased positive and decreased negative self-responding in times of personal struggle [19]. Self-compassion requires being kinder, more supportive and less harshly judgmental toward oneself. It includes a greater recognition of the shared human experience, understanding that all humans are imperfect and have imperfect lives, and being less inclined to feel isolated by one’s imperfection. It requires mindful awareness of personal suffering and ruminating less about the negative aspects of oneself or one’s life experience. The six components of self-compassion are conceptually distinct but still act as a system, tapping into different ways that individuals emotionally react to suffering (with kindness or judgment), cognitively understand their predicament (as part of the human experience or as isolating), and pay attention to pain (in a mindful or over-identified manner)[20]. Self-compassion has been introduced as an important protective variable against the stresses experienced by HCWs [21]. Self-compassion has been a major topic of interest in support/training programs for HCWs in France during the pandemic [22].

Although research on the relationship between self-compassion and PTSD is still new, the body of literature is growing. Various studies conducted on different samples have demonstrated that the components of self-compassion are negatively related to PTSD symptoms [23–26]. In fact, the stronger the components of self-compassion are in a person, the less likely they are to suffer from PTSD symptoms [20].

Therefore, given that HCWs are one of the populations most vulnerable to PTSD, the present study was conducted to investigate two influential psychological factors (self-compassion and perceived social support and their components) and the demographic factors contributing to PTSD. Better knowledge of these contributors may reduce the PTSD burden in HCWs during the ongoing COVID-19 pandemic [7]. The centers selected for the current research are educational health centers affiliated to Tabriz University of Medical Sciences. These hospitals were selected because they are the main referral centers for COVID-19 in the province, and the HCWs working in them, especially the emergency room staff, have experienced the greatest burden of care possible during the pandemic.

## Methods

### Study design and procedure

This descriptive-correlational study was conducted in 2020–2021 in Tabriz and was reviewed and approved as a research project by the Research Ethics Committee of Tabriz University of Medical Sciences. We confirm that all methods were performed in accordance with the relevant guidelines and World Medical Association Declaration of Helsinki. Also, we confirm that written informed consent was obtained from all staff who participated in the study. After receiving the research ethics code, arrangements were made with the intended health centers and hospitals to obtain the required permits. Then, after obtaining the written consent of the participants, the questionnaires were distributed among them. All the questionnaires used in this study were valid Persian translations of pencil-and-paper scales originally published in English. The study lasted eight months (October 2020 to June 2021) from the time of obtaining the permits to the time of writing the manuscript. Two consecutive weeks were collected data, which coincided with the third and fourth peaks of the COVID-19 pandemic in Iran.

The researchers presented to the ward in different shifts and personally handed the questionnaires to the staff, who were asked to fill them out anonymously and place them in a dedicated box at the nursing station.

### Study sites

This study was performed in two main hospitals affiliated to Tabriz University of Medical Sciences, including Imam Reza Hospital and Sina Hospital.

Imam Reza Hospital is the largest referral hospital in the northwest of Iran, which is located in the city of Tabriz, East Azerbaijan Province. The hospital has 804 active beds and 23 wards, 11 ICUs, and eight operating rooms. Since the onset of the pandemic, 20 wards, eight ICUs, and five operating rooms in this hospital have been allocated to COVID-19 patients. The average number of patients presenting to the emergency room was 11,225 and the average number of hospitalizations 2749 per month. The hospital has treated 36,375 COVID-19 patients since the beginning of the pandemic.

Sina Hospital is the second largest hospital in Tabriz and is a referral center for burn and poisoning events in northwestern Iran. The hospital consists of 374 active beds with 18 wards, 17 ICUs, and three operating rooms. Since the onset of the pandemic, 15 wards, five ICUs, and two operating rooms have been allocated to COVID-19 patients. The average number of patients admitted to the emergency room of this hospital is 5000 per month and the average number of hospitalizations 1500.

### Participants

Census sampling was used to select all emergency personnel staff of Imam Reza and Sina hospitals, two referral COVID-19 educational and health centers in Tabriz, Iran.

Inclusion criteria:

- Working in medical departments as a student, doctor, nurse or service personnel for at least one year.
- Working in treatment centers for COVID-19 from at least three months before starting the present study.

Exclusion criteria:

- Having a severe psychiatric illness that, according to the existing literature, could have a significant impact on the development of PTSD [27]. None of the participants were excluded due to this criterion.
- Being infected with COVID-19. Because infection can cause PTSD symptoms on its own [28] and may interfere with our study outcome, the participants with a history of infection were excluded. Twelve participants were excluded from the study due to getting infected during the data collection stage.

### Statistical analysis

Data were analyzed in IBM SPSS Statistics Version 22. The results were reported as frequency (percentage) for the qualitative variables and median (IQR or Interquartile Range) for the quantitative data. The Kolmogorov–Smirnov test was used to test the normality of the distribution of the quantitative variables. Other inferential statistics, including Mann–Whitney’s U test, the Kruskal–Wallis test, Spearman’s correlation coefficient (due to the non-normal nature of data distribution) and the linear multiple regression, were used to analyze the data and to test the relationship between the variables. P < 0.05 was taken as the level of statistical significance. More precisely, we used Kruskal–Wallis and Mann–Whitney’s U test to compare PTSD among groups with different demographic characteristics, and Spearman’s correlation was used to examine the relationship between PTSD and other variables, while multiple regression was used to obtain partial effects after adjusting for other variables [29].

## Measures

Four scales were used for measuring the study variables.

### Researcher-made demographic checklist

This questionnaire collected participants’ personal and occupational demographic information. This checklist includes age, marital status (single/married), gender (female/male), level of education (high school diploma, bachelor’s degree or undergraduate student, master’s degree or postgraduate student, PhD or doctoral student, assistant specialist, and specialist), job (emergency staff, including supervisor nurse, head nurse, nurse, nursing student, specialist, medical intern, resident, and hospital staff or hospital service personnel), work experience (1 to 5 years/ 5 to 10 years/ 10 to 20 years/ more than 20 years) and a history of infection with COVID-19.

### PCL-5

Post-traumatic stress disorder (PTSD) was assessed by the PTSD Checklist for DSM-5 (PCL-5). PCL-5 is a self-report measure including 20 items that correspond directly to the DSM-5 PTSD. Each item shows the severity of a special symptom, rated on a five-point Likert scale from 0 (not at all) to 4 (extremely) during the previous month. The score of each symptom cluster is calculated as the sum of the corresponding items. PCL-5 can determine a provisional diagnosis in two ways: (a) The presence (endorsed as 2 or greater) of at least one re-experiencing symptom (i.e. one Criterion B item—questions 1–5), one avoidance symptom (Criterion C item—questions 6–7), two negative alterations in cognition or mood symptoms (Criterion D items—questions 8–14) and two arousal symptoms (Criterion E items—questions 15–20), and (b) The sum of the total score over cut point score of 47 point [30]. A cut-off point of 47 has been used to detect PTSD [31]. The tool’s Cronbach's alpha was 0.95 for the English version and 0.94 for the French version. The convergent validity of the English version of the tool was reported as 0.89. In Iran, for the Persian version, Cronbach's alpha coefficient was reported as 0.92 for the whole scale and 0.90, 0.67, 0.74, 0.70 and 0.85 for the factors including disturbance, avoidance, negative alterations in mood, arousal and restlessness and emotional numbness, respectively[31]. Cronbach's alpha of this scale in the current study was 0.96.

### Multidimensional Perceived Social Support Scale (MSPSS)

This scale was designed by Zimet et al. and includes 12 items. It measures perceived social support from three sources: Family, friends and significant others. This scale measures the respondent’s perceived social support in all three areas with seven options, from strongly disagree to strongly agree. To obtain the total score of this scale, the scores of all the items are added together and divided by their total number (12). The score of each subscale is obtained from the sum of the scores in that subscale divided by the number of items in the subscale (4)[32]. Zimet et al. reported the validity and reliability of this scale as desirable. In the Iranian population, the reliability of the scale was reported as Cronbach's alpha coefficients of 0.86, 0.86 and 0.82, respectively, for social support received by family, friends and significant others [33]. Cronbach's alpha of this scale in the current study was 0.91.

### Self-Compassion Scale (SCS)

This scale was designed in 2011 and includes 12 items scored on a 5-point Likert scale from 1 (almost never) to 5 (almost always). The scale has three binary components in six subscales, including self-kindness vs. self-judgment, mindfulness vs. over-identification, and common humanity vs. isolation [34]. In the present study, we used the validated Persian version of this scale, as assessed by Shahbazi et al. in a study titled "Confirmatory factor analysis of the Persian version of the self-compassion rating scale-revised. "and the Cronbach's alpha was reported as 0.91 for the whole scale and 0.83, 0.87, 0.91, 0.88, 0.92, and 0.77 for the subscales of self-kindness, self-judgment, common humanity, isolation, mindfulness and extreme imitation, respectively [35]. Cronbach's alpha of this scale in the current study was 0.67.

## Results

### Demographic characteristics and descriptive statistics

In this section, our aim was to describe the characteristics of the participants and the relationship between the demographic indicators and other variables***.***

A total of 250 pen-and-paper questionnaires were distributed among the participants, and 222 of them successfully returned the questionnaires and entered the study based on the eligibility criteria. The response rate was 88.8%. Data were thus collected from 222 emergency staff working in two referral COVID-19 educational health centers affiliated to Tabriz University of Medical Sciences (emergency staff, including supervisor nurse, head nurse, nurse, nursing student, specialist, medical intern, resident, and hospital staff personnel or hospital service personnel).

The mean age of the participants was 31.24 ± 6.85 years with a minimum of 21, a maximum of 55, and a median of 29 years. Female participants comprised 53.4% of all the participants. As for education, 72.8% of the participants had bachelor’s degrees. Also, 57.5% of the participants were nurses. About 77% of the participants had less than ten years and 23% more than ten years of experience as HCWs (Table 1: Demographic Information). The participants were recruited six months after the first case of hospitalization for COVID-19 from two hospitals in Tabriz based on the inclusion and exclusion criteria. The cut-off point of 47 was used to determine the presence or absence of PTSD, which was found to be present in 18 participants (8.8%) and absent in 186 participants (91.2%). PTSD was non-existent/minimal in 55 people (27%), mild in 56 people (27.5%), moderate in 59 (28.9%), severe in 56 (12.7%), and very severe in eight (3.9%). There was a statistically significant relationship between the age of the participants and the overall score of PTSD, as PTSD increased with age (*r* = 0.17, *P* = 0.034).

**Table 1:** Demographic characteristics of study participants

	**Variable**	**Number**	**Percentage**
**Gender**	**Female**	118	53.2
	**Male**	104	46.8
**Marital Status**	**Single**	100	45
	**Married**	122	55
**Education**	**Diploma**	25	12.1
	**Bachelor or** **Undergraduate** **student**	150	72.8
	**Master or graduate student**	20	9.7
	**PhD or** **PhD Student**	5	2.4
	**Resident**	4	1.9
	**Specialist**	2	1
**Job**	**Doctors**	10	5
	**Nurses**	121	60.5
	**Service personnel**	69	34.5
**Work Experience**	**1–5 years**	109	53.7
	**5–10 years**	47	23.2
	**10–20 years**	42	20.7
	** > 20 years**	5	2.5

Table 2 shows the descriptive statistics related to PLC-5, SCS and MSPSS scores and the scores of their subscales. The comparison of the median total score of PTSD between the different classes of each of the variables related to demographic characteristics showed no statistically significant relationship with gender (*P* = 0.774), marital status (*P* = 0.22), education (*P* = 0.328), job (*P* = 0.272) and work experience (*P* = 0.094). Also, based on the categories of PTSD, PTSD severity was not significantly related to gender and marital status (*P* = 0.836 and *P* = 0.383; Table 1 in supplemental file).

**Table 2:** Descriptive statistics of the PTSD, Self-Compassion, and Multidimensional Perceived Social Support scale scores and the scores of their subscales

Variable	Number	Minimum	Maximum	*First Quarter*	Midian	*Third Quarter*
PTSD	204	0	78	10	19	33
Criterion B (Re-experiencing)	217	0	4	0.4	1.2	2
Criterion C (Avoidance)	216	0	4	0.5	1.25	2
Criterion D (Negative alterations in cognition and mood)	212	0	4	0.25	0.75	1.75
Criterion E (Hyper-arousal)	214	0	4	0.25	1	1.75
Criterion F	214	0	4	0.4	0.8	1.6
Self-compassion	210	12	54	32	36	39
Self-kindness	219	2	10	6	7	8
Self-judgment	221	2	10	4	5	6
Mindfulness	220	2	10	5	6	8
Over-Identification	219	2	10	4	6	7
Common Humanity	219	2	10	5	6	7
Isolation	221	2	10	4	5	7
Multidimensional Perceived Social Support	211	2	7	4.42	5.42	6
Family	217	1	7	5	6	6.75
Friends	217	1	7	3.75	4.75	5.75
Significant others	218	1	7	4.44	5.5	6.5

### Associations

Table 2 in supplemental file shows the relationship of the overall PTSD with SCS and MSPSS and their subscales, and Table3 shows the relationship between PTSD and the demographic variables and the MSPSS, SCS and their subscales.

**Table 3:** The relationship of PTSD with the demographic variables and the self-compassion and perceived multidimensional social support scale and their subscales

Variable	Category	β	95%CI	P
**Age (years)**		0.901	0.19 – 1.61	0.013^*^
**Gender**	**Female**	1.410	-4.24 – 7.06	0.622
	**Male**	Ref. Category		
**Marital Status**	**Single**	0.492	-5.51 – 6.49	0.871
	**Married**	Ref. Category		
**Education**		-3.496	-9.625 – 2.63	0.261
**Job**	**Doctors**	-9.201	-33.17 – 14.77	0.448
	**Nurses**	1.579	-5.08 –8.24	0.639
	**Service personnel**	Ref. Category		
**Work Experience**	**1–5 years**	6.061	-15.21 – 27.33	0.573
	**5–10 years**	2.853	-16.99 – 22.70	0.776
	**10–20 years**	-1.099	-17.99 – 15.80	0.898
	** > 20 years**	Ref. Category		
**Direct Contact With a COVID-19 Patient**	**Yes**	-0.048	-9.24 – 9.14	0.992
	**No**	Ref. Category		
**Self-Kindness**		-0.799	-2.63 – 1.03	0.388
**Self-Judgment**		2.406	0.504—4.31	0.014^*^
**Mindfulness**		-0.036	-1.945 – 1.87	0.970
**Over-Identification**		-0.561	-1.996 – 0.87	0.440
**Common Humanity**		0.270	-1.431 – 1.97	0.754
**Isolation**		2.493	0.908 – 4.08	0.00^*^
**Family**		-0.442	-3.053 – 2.17	0.738
**Friends**		-0.283	-2.264 – 1.70	0.778
**Significant Others**		1.108	-1.219 – 3.43	0.347

*R*-square = 0.34, Adjusted *R*-square = 0.25, * Significant at the level of 0.05

The relationship between the overall score of PTSD and the SCS and MSPSS and their subscales was also examined using Spearman’s correlation coefficient, and a significant relationship was found between the SCS score and the overall score of PTSD (*r* = 0.27, *P* < 0.001; Fig. 1 in supplemental file).

There was a statistically significant inverse relationship between the score of the self-kindness.

subscale and the overall score of PTSD (*r* =—0.19, *P* = 0.006; Fig. 2 in supplemental file).

There was a statistically significant relationship between the score of self-judgment and the overall score of PTSD (*r* = 0. 36, *P* < 0.001; Fig. 3 in supplemental file).

There was a statistically significant relationship between the score of over-identification and the overall score of PTSD (*r* = 0.15, *P* = 0.031; Fig. 4 in supplemental file).

There was a statistically significant relationship between the score of isolation and the overall score of PTSD (*r* = 0.44, *P* < 0.001; Fig. 5 in supplemental file).

No correlation was found between the overall score of PTSD and the rest of the subscales of the SCS and MSPSS (*P* < 0.05; Table 2 in supplemental file).

### Multiple regression analysis

In this section, although our goal was to examine the relationship between the variables, multiple regression analysis was used to obtain partial effects after adjusting for the other variables.

The results of the linear multiple regression analysis showed that if the demographic variables are controlled, the correlation between the subscales of SCS (self-judgment and isolation) and the PTSD score is significant. In other words, if the other regression model variables are kept constant, one unit of increase in the self-judgment score increases the overall PTSD score by 2.41 points. Also, by keeping the other variables of the regression model constant, one unit of increase in the isolation score increases the overall score of PTSD by 2.49 units. In this study, a significant relationship was observed between participants’ age and their overall score of PTSD, such that with the other variables kept constant, increasing age by one year increases the overall score of PTSD by 0.90 units. Also, a significant relationship was observed between participants’ education and their overall score of PTSD, such that with the other variables kept constant, increasing education by one level decreased the overall score of PTSD by 3.50 units (Table 3).

## Discussion

In the last two years, COVID-19 has been inflicting global communities as a life-threatening disease. Emergency room staff, who have always been exposed to a variety of stressors in the workplace, have been increasingly exposed to physical and psychological problems during the COVID-19 pandemic as the first-line healthcare workers. They take care of patients in different work shifts around the clock, with long work hours, sleep deprivation, and workplace issues. Furthermore, having to maintain a distance from their family causes various problems for them. Since the beginning of the COVID-19 pandemic, various studies have been conducted in Iran on the psychological effects of the disease on HCWs, and some of them have reported a high prevalence of PTSD in this group [36, 37]. Among the studies conducted in Iran, a review study showed that the greatest psychological effect of COVID-19 in HCWs is observed in the form of post-traumatic stress disorder, depression, anxiety, stress, sleep disorders and anger [36].

The present study aimed to investigate the relationship between PTSD and demographic and psychological variables such as age, gender, education, occupation, self-compassion and perceived social support. According to the results, of the six components of bipolar self-compassion, kindness was inversely related to the overall PTSD score while self-judgment, isolation and over-identification were all related to the PTSD score directly. Although some other research has shown the role of occupation, the variables of marital status, age, gender, quarantine, stigma, previous psychiatric disorders, isolation, and being survivors of the same prevalence (SARS and MERS) also emerged as strong risk factors for PTSD [7]. In the present study, age and education were the demographic factors related to the overall PTSD score when other variables were kept constant. Meanwhile, the findings regarding gender and marital status were inconsistent with the results reported by Carmassi et al. and Cunha et al. [7, 38].

Age has been suggested as an important risk factor for the severity and prognosis of COVID-19 and its associated mortality. Access to such news and information may affect people’s perception of danger, and older people think they are at a higher risk than others. One possible explanation for the relationship observed between education and COVID-19 symptoms can be the greater information that more educated people gather about the disease. As a result, PTSD symptoms were lower among physicians and specialists than among other personnel. A study in Brazil also found that demographic characteristics such as age, gender, employment status, and social support affect the experience of mental suffering in healthcare workers during the COVID-19 pandemic [38].

One of the hypotheses confirmed in the present study was an inverse relationship between self-compassion and PTSD. To further justify the present findings, it can be argued that people who have high self-compassion do not exacerbate the inevitable suffering with ineffective strategies such as self-judgment, isolation, and over-identification. They accept that these events and sufferings are inevitable and that all human beings will experience them more or less. Conversely, those with high self-judgment blame themselves for the weaknesses and intensify the suffering and stress caused by the trauma instead of acknowledging their weaknesses and focusing on their ability to cope with trauma. Also, people with high scores in the isolation index think that they are alone in enduring this suffering and imagine that this trauma is something that solely affects *them*, and this feeling of loneliness and isolation makes the burden of enduring trauma heavier than before. Most of these people do not let other people join them in enduring suffering. These results were in line with previous research on the relationship between PTSD and self-compassion [20, 25, 39]. Also, other studies have shown that self-compassion is associated with a reduction in psychological pathologies and an increase in psychological well-being. In fact, the positive indicators of self-compassion (self-kindness, mindfulness, and common humanity) are negatively correlated with a psychological pathology, while the negative indicators of self-compassion (self-judgment, over-identification, and isolation) are positively correlated with a psychological pathology and make the subject vulnerable to mental health problems [39–42].

The relationship of self-judgment (one of the components of self-compassion (and PTSD was also confirmed in the present study. Self-judgment has been directly linked to psychopathological symptoms [19]. Self-judgment is criticism of one's own shortcomings and inadequacies. Acknowledging that all human-beings are flawed and mistaken and tend to engage in unhealthy behaviors is a hallmark of being a human, as in contrast to the isolation component that was associated with the PTSD score in the present study. Instead of watching themselves consciously and looking at their experience from a higher perspective that leads to realism and comprehensiveness, people may judge themselves and feel isolated as individuals during times of suffering and in challenging situations. Self-judgment, isolation, and over-identification in experiencing difficult situations, such as the COVID-19 pandemic, can put people at a greater risk of PTSD symptoms. People with high self-judgment scores feel incapable in the face of risks and trauma; they believe that cannot cope with such problems.

Evidently, emergency medical staff are at a higher risk due to their direct exposure to many patients with the coronavirus, and their anxiety and concerns about the disease as a trauma are therefore higher than those of the general public. Nonetheless, the reaction to trauma is not the same as the public in medical community members, and there are probably a variety of psychological and social factors involved in determining the degree of anxiety about infection in this group. The results of this study show that the components of self-compassion are related to the symptoms of PTSD and explain why some emergency medical personnel show more severe symptoms.

Given the prolongation of the COVID-19 pandemic and the consequent long-term involvement of HCWs with the disease, promoting knowledge about the psychological effects of this pandemic is very effective in designing appropriate interventions for this group. Disorders such as PTSD can have serious long-term effects on the personal life and professional services of HCWs. Therefore, relevant organizations should design special intervention packages for at-risk HCWs based on research evidence that shows the risk factors for COVID-19. As several studies have shown, a variety of acceptance and commitment-based treatment (ACT) protocols can be effective in treating PTSD. Considering the special conditions of the HCWs during the pandemic and according to the effective components found in the present study, fit protocols can be designed for this specific group [43, 44].

## Conclusion

The aim of this study was to investigate the relationship between PTSD and possible related psychological components such as self-compassion and perceived social support. The findings showed that age, education, self-judgment, isolation and over-identification were associated with PTSD score, and also there was a statistically significant inverse relationship between the score of the self-kindness subscale and the overall score of PTSD in the emergency staff. These results suggest that some psychological and demographic factors can play the role of protective factors or risk factors in critical situations such as pandemics among emergency staff.

Given the uncertainty about the end of the COVID-19 pandemic and its control, and given the psychological problems the current situation has imposed on people globally, especially medical staff, more research should be carried out on COVID-19-related psychological trauma, with a special emphasis on treatment staff. By identifying the predisposing factors of the studied psychological pathology, this research can be applied in clinical practice and provide useful information for designing intervention protocols.

## Limitations

One of the limitations of the present study was limiting HCWs to emergency room staff, which means that the results should be generalized with caution. Also, this research was conducted in Tabriz and the mother tongue of the participants was Turkish, and although the national and educational language of Iran is Persian and all the participants were fluent in Persian, this issue could be a limitation of the study and could affect the responses to the questionnaires.

## Supplementary Information

**Additional file 1: Table 1.** Comparison of PTSD severity between the gender and marital status.** Table2.** The relationship of the overall PTSD with self-compassion and perceived multidimensional social support and their subscales. **Figure1.** The relationship between the self-compassion score and the overall PTSD score. **Figure 2.** The correlation between the self-kindness subscale score and the overall PTSD score. **Figure3.** The correlation between the self-judgment score and the overall PTSD score. **Figure 4.** The correlation between the extreme replication subscale score and the overall PTSD score. **Figure 5.** The correlation between the isolation subscale score and the overall PTSD score.

## Data Availability

The datasets used and/or analyzed during the current study are available from the corresponding author on reasonable request.

## Abbreviations

COVID-19: Coronavirus Disease
HCWs: Healthcare Workers
MERS: Middle East Respiratory Syndrome
MSPSS: Multidimensional Perceived Social Support Scale
PTSD: Post-Traumatic Stress Disorder
PCL-5: PTSD Checklist for DSM-5
SARS: Severe Acute Respiratory Syndrome
SCS: Self-Compassion Scale
WHO: World Health Organization
