# Supplementary material for: Prediction of PTSD related to COVID-19 in emergency staff based on the components of self-compassion and perceived social support
Source: BMC Psychiatry. 2022 May 31;22:368. doi: 10.1186/s12888-022-04017-8 (PMC9154198; doi:10.1186/s12888-022-04017-8)
Supplement: Supplementary file 1 — Additional file 1: Table 1. Comparison of PTSD severity between the gender and marital status. Table2. The relationship of the overall PTSD with self-compassion and perceived multidimensional social support and their subscales. Figure1. The relationship between the self-compassion score and the overall PTSD score. Figure 2. The correlation between the self-kindness subscale score and the overall PTSD score. Figure3. The correlation between the self-judgment score and the overall PTSD score. Figure 4. The correlation between the extreme replication subscale score and the overall PTSD score. Figure 5. The correlation between the isolation subscale score and the overall PTSD score. [file 12888_2022_4017_MOESM1_ESM.docx]

**Table 1: Comparison of PTSD severity between the gender and marital status**

|  |  | Severity of PTSD | | | | | P^*^ |
| --- | --- | --- | --- | --- | --- | --- | --- |
|  |  | **Non/minimal** | **Mild** | **Moderate** | **Sever** | **Very sever** |  |
| Gender | **Female** | 29 (26.6) | 29 (26.6) | 31 (28.4) | 14 (12.8) | 6 (5.5) | 0.836 |
|  | **Male** | 26 (27.4) | 27 (28.4) | 28 (29.5) | 12 (12.6) | 2 (2.1) |  |
| marital status | **Single** | 29 (31.2) | 28 (30.1) | 21 (22.6) | 12 (12.9) | 3 (3.2) | 0.383 |
|  | **Married** | 26 (23.4) | 28 (25.2) | 38 (34.2) | 14(12.6) | 5 (4.5) |  |

^*^Fisher exact test

**Table 2: The relationship of the overall PTSD with self-compassion and perceived multidimensional social support and their subscales**

| PTSD | | Variable |
| --- | --- | --- |
| P-Value | **Spearman’s Correlation Coefficient (r)** | **Variable** |
| 0.034* | 0.168 | **Age** |
| <0.0001* | 0.269 | **Self-compassion** |
| 0.006* | -0.194 | **Self-kindness** |
| <0.0001* | 0.359 | **Self-judgment** |
| 0.527 | -0.045 | **Mindfulness** |
| 0.031* | 0.151 | **Over-Identification** |
| 0.5 | -0.048 | **Common Humanity** |
| <0.0001* | 0.442 | **Isolation** |
| 0.391 | -0.062 | **Multidimensional Perceived Social Support** |
| 0.195 | -0.092 | **Family** |
| 0.69 | -0.028 | **Friends** |
| 0.702 | -0.027 | **Significant others** |

^*^ Significant at the level of 0.05


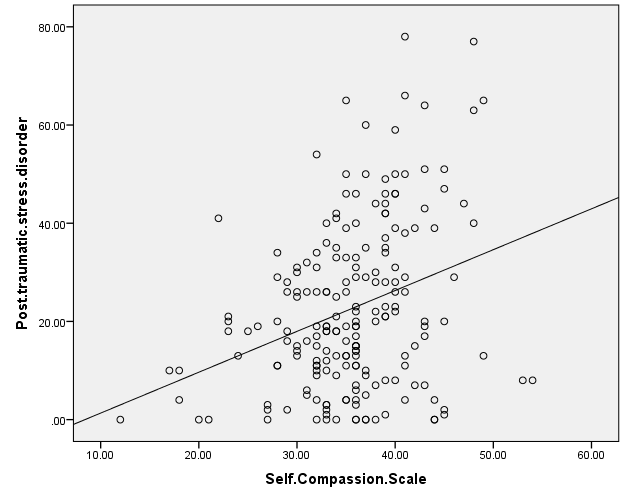


Figure 1: The relationship between the self-compassion score and the overall PTSD score


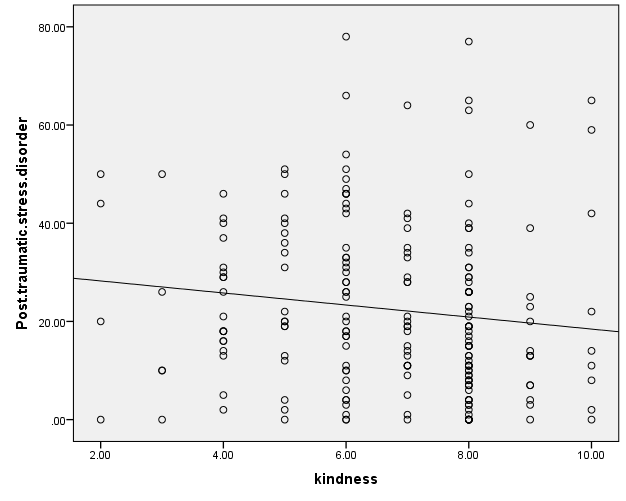


Figure 2: The correlation between the self-kindness subscale score and the overall PTSD score


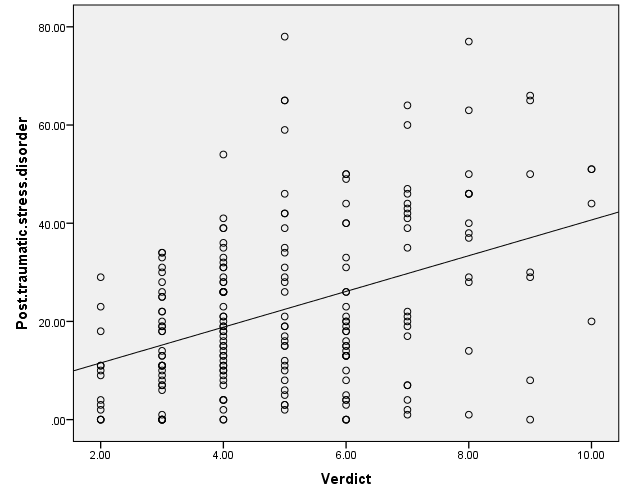


Figure 3: The correlation between the self-judgment score and the overall PTSD score


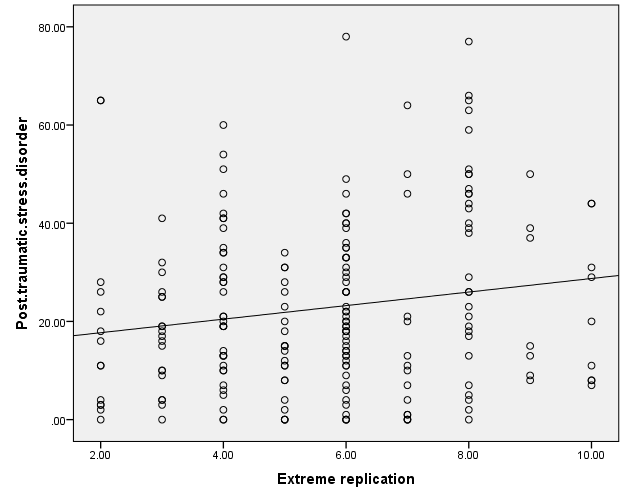


Figure 4: The correlation between the extreme replication subscale score and the overall PTSD score


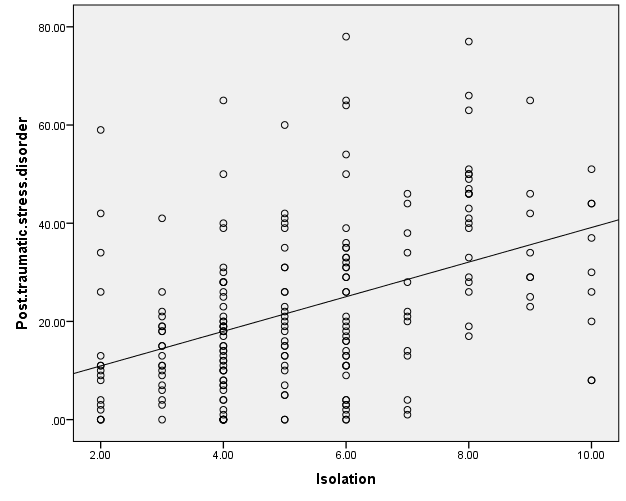


Figure 5: The correlation between the isolation subscale score and the overall PTSD score
